# The Genetic Analysis of an *Acinetobacter johnsonii* Clinical Strain Evidenced the Presence of Horizontal Genetic Transfer

**DOI:** 10.1371/journal.pone.0161528

**Published:** 2016-08-22

**Authors:** Sabrina Montaña, Sareda T. J. Schramm, German Matías Traglia, Kevin Chiem, Gisela Parmeciano Di Noto, Marisa Almuzara, Claudia Barberis, Carlos Vay, Cecilia Quiroga, Marcelo E. Tolmasky, Andrés Iriarte, María Soledad Ramírez

**Affiliations:** 1 Instituto de Investigaciones en Microbiología y Parasitología Médica (IMPaM, UBA-CONICET), Buenos Aires, Argentina; 2 Department of Biological Science, California State University Fullerton, Fullerton, CA, United States of America; 3 Laboratorio de Bacteriología Clínica, Departamento de Bioquímica Clínica, Hospital de Clínicas José de San Martín, Facultad de Farmacia y Bioquímica, Buenos Aires, Argentina; 4 Departamento de Desarrollo Biotecnológico, Instituto de Higiene, Facultad de Medicina, UdelaR, Montevideo, Uruguay; University of Sydney, AUSTRALIA

## Abstract

*Acinetobacter johnsonii* rarely causes human infections. While most *A*. *johnsonii* isolates are susceptible to virtually all antibiotics, strains harboring a variety of β-lactamases have recently been described. An *A*. *johnsonii* Aj2199 clinical strain recovered from a hospital in Buenos Aires produces PER-2 and OXA-58. We decided to delve into its genome by obtaining the whole genome sequence of the Aj2199 strain. Genome comparison studies on Aj2199 revealed 240 unique genes and a close relation to strain WJ10621, isolated from the urine of a patient in China. Genomic analysis showed evidence of horizontal genetic transfer (HGT) events. Forty-five insertion sequences and two intact prophages were found in addition to several resistance determinants such as *bla*_PER-2_, *bla*_OXA-58_, *bla*_TEM-1_, *strA*, *strB*, *ereA*, *sul1*, *aacC2* and a new variant of *bla*_OXA-211,_ called *bla*_OXA-498_. In particular, *bla*_PER-2_ and *bla*_TEM-1_ are present within the typical contexts previously described in the *Enterobacteriaceae* family. These results suggest that *A*. *johnsonii* actively acquires exogenous DNA from other bacterial species and concomitantly becomes a reservoir of resistance genes.

## Introduction

Human infections caused by non- *A*. *baumannii* members of the *Acinetobacter* genus have been recently recognized due to the implementation of new technologies in clinical diagnostic laboratories [1–6]. Forty-seven distinctive *Acinetobacter* species with valid names have been described and more species are identified each year [7, 8]. *Acinetobacter johnsonii* is usually found in the environment and animals [9], it can occasionally colonize human skin [10] and cause clinical infections such as catheter-related bloodstream infections [11] or peritonitis associated with peritoneal dialysis [12].

Although multidrug resistance has not been widespread among non-*baumannii Acinetobacter* species [13], *A*. *johnsonii* strains harboring a variety of β-lactamase genes are being isolated at different geographic locations [12–19]. One such isolate, recently described by Rodriguez et al., is strain (Aj2199) which produces PER-2 and OXA-58 [12]. These findings strongly suggest that *A*. *johnsonii* can acquire exogenous DNA as these genes were previously described in other bacterial genus and species. The detection of β-lactamase genes and other resistance determinants among *Acinetobacter* species demonstrates their potential to acquire and stably maintain resistance determinants.

In this study, we determined the complete genome sequence of the Aj2199 strain. The presence of a variety of traits like antibiotic resistance genes, insertion sequences, phage sequences, and numerous unique genes are indicative of active horizontal genetic transfer (HGT).

## Materials and Methods

### Whole-genome sequence of Aj2199 clinical strain

Genomic DNA was extracted using a MasterPure DNA Purification kit from Epicentre Biotechnologies. Whole-genome shotgun sequencing was performed using Illumina MiSeq- I, with Nextera XT libraries for sample preparation. De novo assembly was performed with SPADES assembler version 3.1.0 [14], using a pre-assembly approach with Velvet [15]. RAST server was used to predict open reading frames [16] and BLAST (version 2.0) software was utilized to confirm the predictions. tRNAscan-SE was used to predict tRNA genes [17]. This Whole Genome Shotgun project has been deposited at DDBJ/ENA/GenBank under the accession LVIB00000000. The version described in this paper is version LVIB01000000.

### Genome Sequences for comparative genomics

All available assemblies of *A*. *johnsonii* strains were downloaded from NCBI via ftp at ftp.ncbi.nlm.nih.gov/genomes/ASSEMBLY_BACTERIA. Other complete genomes, draft genomes and assemblies were also downloaded from NCBI via ftp and used for comparative studies. Up to three randomly selected genomes for each species were included in the analysis in order to avoid over-representation of some species. Reference genomes of the genus: *A*. *baumannii* AYE and *A*. *baumannii* ACICU were also analyzed (See S1 Table for the complete list of the 97 genomes sequences used in this work). Assemblies were annotated by means of RAST server [16] and the SEED source for genome annotations [18] with default parameters. GO ontology annotation was performed by means of HMM profile searches against bacterial EggNog 4.5 database [19] using HMMER 3.1 software, available at http://hmmer.org/.

### Clustering of homologous genes and phylogenetic analysis

Identification of homologous genes among the analyzed genomes was carried out using the OrthoMCL method [20] by means of the get homologous software [21]. Blastp searches were done with a minimum e-value of 1.10^−5^, a minimum identity value of 30% and minimum query coverage of 75%. 301 groups of putative orthologous sequences were identified among genomes of the family *Moraxellaceae* (Class: *Gammaproteobacteria*, Order: *Pseudomonadales*). Protein sequences alignments were done using Clustal Omega v1.2.0 [22]. Phylogenies were inferred using the maximum-likelihood method with an amino acid LG+G (8 categories) model by means of Phyml version 3.1 [23] with 5 random starting trees. The default SH-like test was used to evaluate branch supports in each analysis [24]. Finally, a consensus tree was inferred from the 301 trees using the sumtrees.py script [25]. In the consensus tree, node support represents the number of phylogenies in which certain node appeared.

### Average nucleotide identity

Average nucleotide identity (ANI) between *A*. *johnsonii* Aj2199 and other closely related genomes (all *A*. *johnsonii* strains plus *A*. *lwoffii* WJ10621) was estimated. The ANI was used to delineate species using genome sequence data [26], two genomes displaying an ANI value equal or higher than 95% belong to the same species. Two-way ANI (reciprocal best hits based comparison) was estimated by means of the ani.rb script developed by Luis M. Rodriguez-R and available at enveomics.blogspot.com.

### Genomic comparison, gene content and sequences analysis

Sequence analysis was carried out using BLAST (version 2.0) software (http://www.ncbi.nlm.nih.gov/BLAST/).

ARG-ANNOT and ISfinder softwares were used to identify antibiotic resistance genes and insertion sequences within the genome of Aj2199, respectively [27, 28]. Furthermore, phages and prophages prediction was done using PHAST (PHAge Search Tool) bioinformatic tool [29]. PlasmidFinder was used to detect the presence of *Enterobacteriacea* plasmids[30]. plasmidSPAdes software, which distinguishes plasmid sequences via the read coverage of contigs, was also used [31].

Prediction of small non-coding RNAs was done using RNA families from the Rfam database and the software Infernal [32].

### Other bacterial strains

Four *A*. *johnsonii* strains (Aj205, Aj286, Aj289, Aj306) from the bacterial collection of the Hospital de Clínicas José de San Martín were included in the present study (Table 1). The four strains were identified as *A*. *johnsonii* by MALDI-TOF MS (Bruker Daltonik), *rpo*B amplification, and sequencing.

**Table 1:** Other *Acinetobacter johnsonii* strains included in this study.

			MIC (mg/L)											
Strain	Year	Sample	AMP	SAM	FOX	CTX	CAZ	IMP	MEM	AMK	GEN	CIP	COL	CEF
Aj306	2013	Urine	≤2	≤2	≤4	≤1	≤1	≤1	≤0.25	≤2	≤1	≤0.25	2	≤1
Aj289	2013	BAL	≤2	≤2	≥ 64	4	4	≤1	≤0.25	≤2	≤1	0.5	≤0.5	4
Aj286	2013	Bone	≥ 32	≥ 32	≥ 64	4	2	≤1	≤0.25	≤2	≤1	0.5	≤0.5	4
Aj205	2013	Urine	≤2	≤2	ND	≤1	≤1	≤1	≤0.25	≤2	≤1	ND	≤0.5	≤1

BAL, bronchoalveolar lavage; AMP, ampicilin; SAM, ampicilin/sulbactam; FOX, cefoxitin; CTX, cefotaxime; CAZ, ceftazidime; IPM, imipenem; MEM, meropenem; AMK, amikacin; GEN, gentamicin; CIP, ciprofloxacin; COL, colistin; CEF, cefepime; ND, no determinated.

### General molecular techniques

Total DNA extraction was carried out using Wizard^®^ Genomic DNA Purification Kit according to manufacturer instructions (Promega, Madison, WI). PCR reactions using the extracted DNA as template were carried out to determine the presence of resistance determinants (*bla*_PER-2_, *bla*_OXA-58_, *bla*_TEM-1_, *strA*, *strB*, *ereA*, *sul1*, *aacC2*) previously identified in Aj2199. The reactions were performed using the GoTaq enzyme according to manufacturer’s instructions (Promega, Madison, USA). Plasmid extraction and *repAcil* gene amplification was carried out to search for the presence of the replicase gene of plasmids harboring *bla*_OXA-58_ [33]. Plasmid DNA extraction was performed using the alkaline lysis method as described [34] with or without further purification using the QIAfilter Midi prep Kit (QIAGEN, Hilden, Germany) according to the manufacturer’s recommendations.

Conjugation assays were carried out to determine if *bla*_TEM-1_ and *bla*_PER-2_ are plasmid located. Briefly, Aj2199 and *E*. *coli* J53-2 cells grown with agitation in Luria Bertani (LB) broth were mixed (1:10 donor:recipient) and incubated for 18 h at 30°C. Transconjugants were selected on LB agar supplemented with sodium azide (100 μg/ml) and ampicillin (100 μg/ml). Transformation assays were carried out using as hosts competent *E*. *coli* TOP10 or the naturally competent *A*. *baumannii* A118 [35]. After transformation *E*. *coli* cells were plated on LB-agar containing ampicillin (100 μg/ml) or ceftazidime (4μg/ml). Transformation of *A*. *baumannii* A118 was carried out as previously described [35]. Briefly, fresh LB was inoculated with cells at late stationary phase followed by addition of 200 ng plasmid DNA and incubated for 1 hour at 37°C. Cells were then plated on LB-agar containing ceftazidime (4μg/ml). Electroporation was performed using the *A*. *baumannii* strains A118 and ATCC 17978, and *A*. *baylyi* ADP1 as described before [36]. Cells were plated on LB-agar containing ceftazidime (4μg/ml).

## Results

### Aj2199 genome sequence, features and comparative genomic analysis

The whole genome sequence of Aj2199 has a total of 1,167,708 high quality paired-end reads with an average insertion size of 514 bp [15]. 99.9% of the generated reads were mapped, resulting in mean nucleotide coverage of 65X (and a k-mer coverage of 29.9). Corrected reads show an average length of 251 bp.155 contigs larger than 500 bp were assembled, comprising 3,803,969 bp. The draft genome has a N50 contig size of 70,198 bp with a maximum contig length of 286,583 bp and a G+C content of 41.4%. A total of 3,737 open reading frames were predicted using the RAST server [16]. Using tRNAscan-SE, a total of 114 tRNA genes were identified [17] (Table 2).

**Table 2:** General features of the *A*. *johnsonnii* Aj2199 genome.

Features	Chromosome
Total number of base pairs	3,803,969
G+C content (%)	41.4%
Number of possible ORFs	3,737
Number of tRNA genes	114
Number of Insertion Sequences (ISs)	45
Number of prophages	4

Prophages include partial and complete sequences.

**Fig 1:**
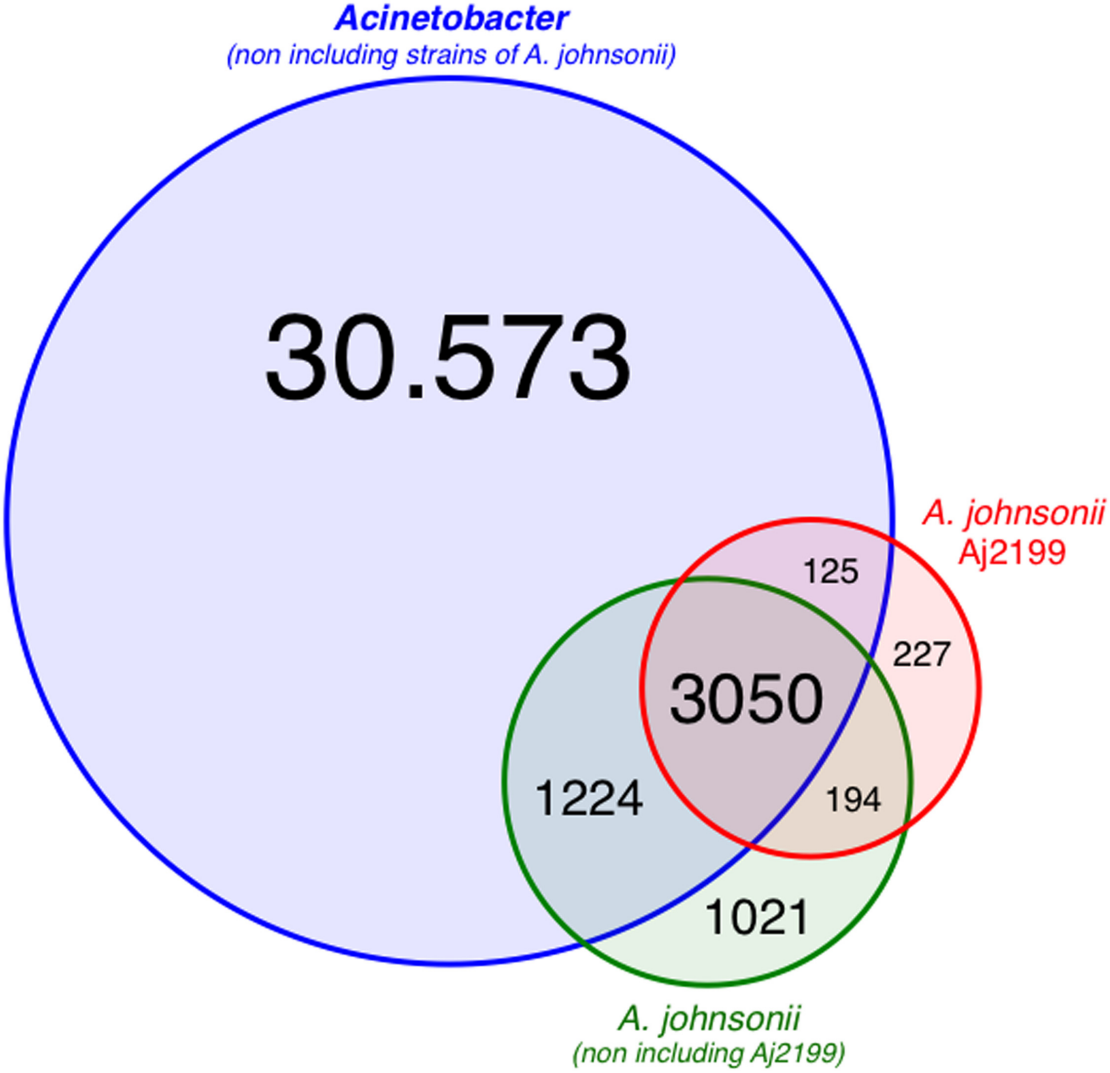
Comparative analysis of the gene families found within the 79 *Acinetobacter* genomes. Venn diagram representing: i) shown in blue is the number of gene families found in the pangenome of *Acinetobacter*, except *A johnsonii* strains, ii) green corresponds to the pangenome of *A*. *johnsonii*, except Aj2199 strain, and iii) in red all gene families found in Aj2199 genome are shown.

We performed a genome comparison between Aj2199 and 79 *Acinetobacter* genomes available in the GenBank and identified 36,414 homologous gene families. The data could be interpreted as a rough estimation of the pangenome of *Acinetobacter*. Minimum values of 30% amino acid identity and 75% coverage was set for grouping genes in homologous families. Among the analyzed genomes, a total of 1,442 gene families were predicted as unique to *A*. *johnsonii* strains (S2 Table), including Aj2199. Homologous identification suggests that more than 63% of the gene families exclusively found in *A*. *johnsonii* are strain-specific, which means that they are only found in one genome. On the other hand, only nine gene families are conserved among the strains and not found in any other analyzed *Acinetobacter* genome. These nine gene families may represent a molecular signature of the species (S2 Table). There are 227 gene families comprised of 240 genes distributed within 84 contigs, that are exclusively found in strain Aj2199 (Fig 1). 161 genes were annotated as hypothetical proteins and 24 as mobile elements. Among the other 55 unique genes in Aj2199 we found genes coding for antibiotic resistance determinants, DNA binding and integration proteins, integral component of membrane, transferases, and phage proteins (S2 Table). Some genes coding for proteins related to ion transport and regulation were found in this genome. Interestingly, genes coding for resistance to toxic metal ions were also found. Finally an aquaporin protein coding gene (homologous to *aqpZ*), two pilus biogenesis proteins (PilO-M) and two glutathione S-transferases (EC: 2.5.1.18), among others were also present (S2 Table).

Assembled contigs, comprising 246.615bp, were predicted as putative plasmid sequences by plasmidSPAdes software [31]. These sequences were re-annotated resulting in 225 coding sequences. All of them were already annotated as part of the Aj2199 genome. Among them we found seven genes that are part of toxin-antitoxin systems, six genes part of restriction modification systems, ParA and ParB partitioning proteins, at least 20 metal resistance genes and four cation transporters (S3 Table). Note that any of the antibiotic resistance genes described in this work were predicted to be located in plasmids according to the plasmidSPAdes prediction. The role and functional implications of these genes will be further investigated in the future.

**Fig 2:**
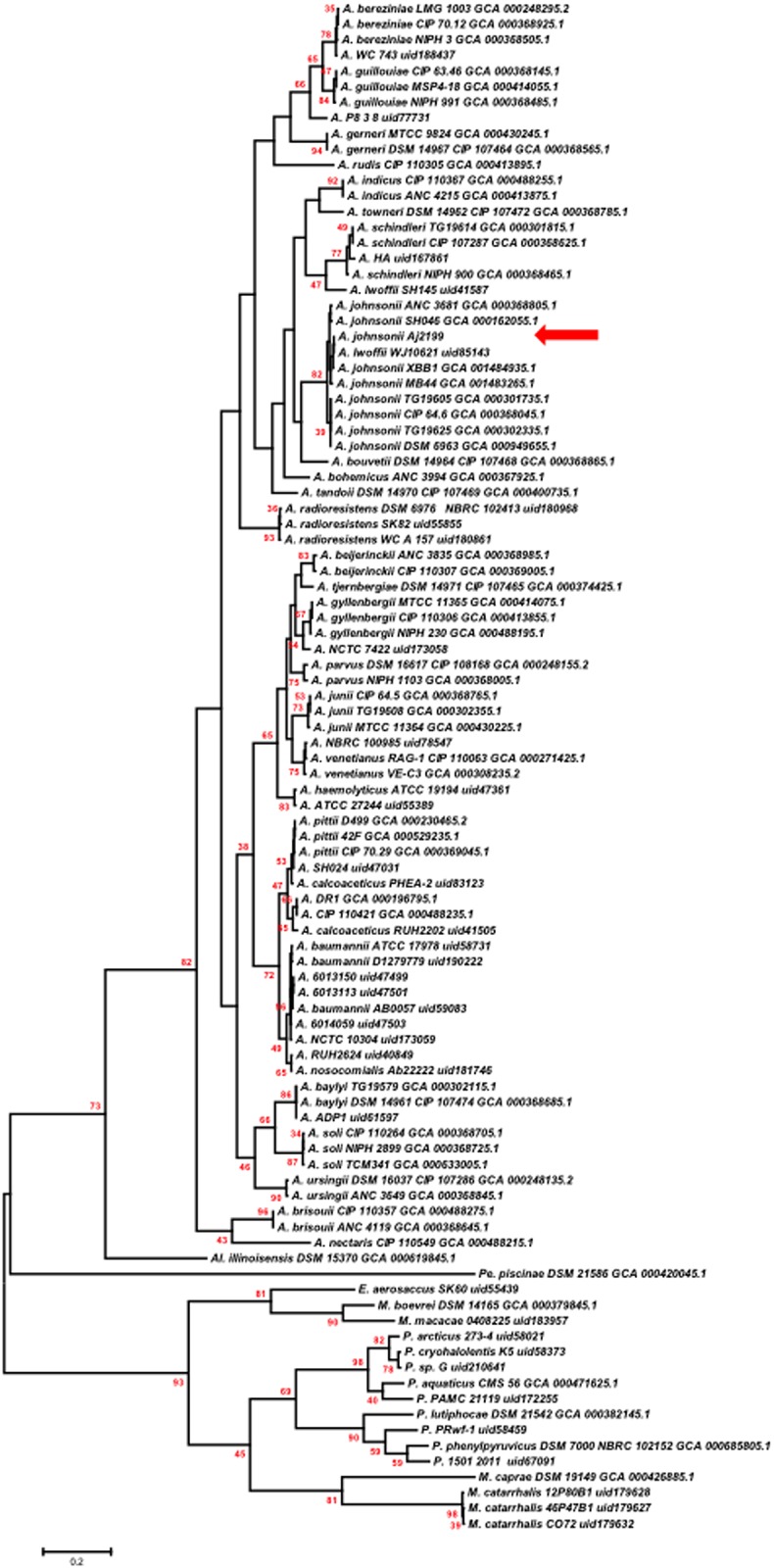
Consensus phylogenetic tree obtained for 98 complete genomes of the *Moraxellaceae* family plus Aj2199 strain. Phylogenetic trees were inferred for 301orthologous protein sequences using the maximum-likelihood method with an amino acid LG+G model. The default SH-like test was used to evaluate branch supports in each case. The consensus tree was inferred from the 301 phylograms, considering only nodes equal or above 50%. Note that node values represent the number of orthologous protein analysis that support a certain group and that node support values bellow 33% are not shown in the Fig The tree was arbitrarily rooted using the mid-point rooting method. A red arrow indicates the position of Aj2199 strain in the tree. In the phylogeny *A*. indicates *Acientobacter*, *M*. indicates *Moraxella*, E. indicates *Enhydrobacter*, P. indicates *Psychrobacter*, Al. indicates *Alkanindiges* and Pe. Indicates *Perlucidibaca* genus.

Further analysis of the genomes revealed the presence of 301orthologs among 98 complete selected genomes of the *Moraxellaceae* family, 97 from the NCBI database plus Aj2199 (S1 Table) that were used for phylogenetic analysis. The 301 orthologous sets were aligned and then a Maximum Likelihood phylogenetic tree was built for each alignment. The 301 phylograms, considering nodes with significant statistical support (SH-like test > 50%), were merged into a consensus tree shown in Fig 2. The resulting phylogeny clustered Aj2199 with *A*. *johnsonii* strains with 82% node support, which indicates that the node appears in 82% of the total phylograms. We found that the WJ10621 strain, reclassified as *A*. *johnsonii* considering the whole genome comparison, is the closest strain to Aj2199. The two-way average nucleotide identity (ANI) among closely related strains strongly supported the phylogenetic results and the 16S *rDNA* sequence analysis. The ANI between Aj2199, WJ10621 and the other *A*. *johnsonii* strains was above 96% (S4 Table). In summary, the estimated two-way ANI values suggest that all the *A*. *johnsonii* genomes grouped as a monophyletic cluster belong to the same species, which is comprised of SH046, ANC_3681, TG19625, CIP64.6, TG19605, DSM_6963, MB44, XBB1, Aj2199 and also WJ10621.

### Genetic context and description of a new variant of *bla*_OXA-211_ in *A*. *johnsonii*

As was previously reported by Perichon *et al* 2014, OXA-like genes were found frequently in *Acinetobacter* species. In *A*. *johnsonnii* three variant of *bla*_oxa-211_, so called *bla*_oxa-211_, *bla*_oxa-280_ and *bla*_oxa-281,_ were described [37].

The genome of Aj2199 contains a new variant of the chromosomally encoded oxacillinase OXA-211 possessing 10 amino acid substitutions compared to the formerly defined OXA-211 (GenBank accession number AEV91550). This *bla*_OXA,_ now called *bla*_OXA-498_, has a 95% nucleotide identity to previously described OXA genes, such as *bla*_OXA-211_ (GenBank accession number KF453234), *bla*_OXA-212_ (GenBank accession number JN861780), and *bla*_OXA-309_ (GenBank accession number HF947514), all of which are found in *A*. *johnsonii* strains. Furthermore, *bla*_OXA-498_ has up to a 96% identity with other *bla*_OXA-211_ variants such as those in *A*. *johnsonii* strains CIP 64.6 (GenBank accession number APON01000041) and ANC 3681 (GenBank accession number APPZ01000001). A comparison using BLAST between OXA sequences of other *Acinetobacter* species shows that *bla*_OXA-498_ has up to a 72% identity, which indicate that these OXA variants are unique to *A*. *johnsonii*. Located upstream of *bla*_OXA-498_ in Aj2199, we found *dnaK*, *grpE*, hypothetical protein (HP) gene, *tolA* and *estA* genes. Among the identified genes, *dnaK*, *grpE*, and *estA* are responsible for the coding of a chaperone, co-chaperone, and esterase A protein, respectively. A similar genetic context lacking the presence of an upstream OXA gene was described in other *Acinetobacter* sp. (GenBank accession numbers CP003847, CP002080, CP002177).

**Fig 3:**
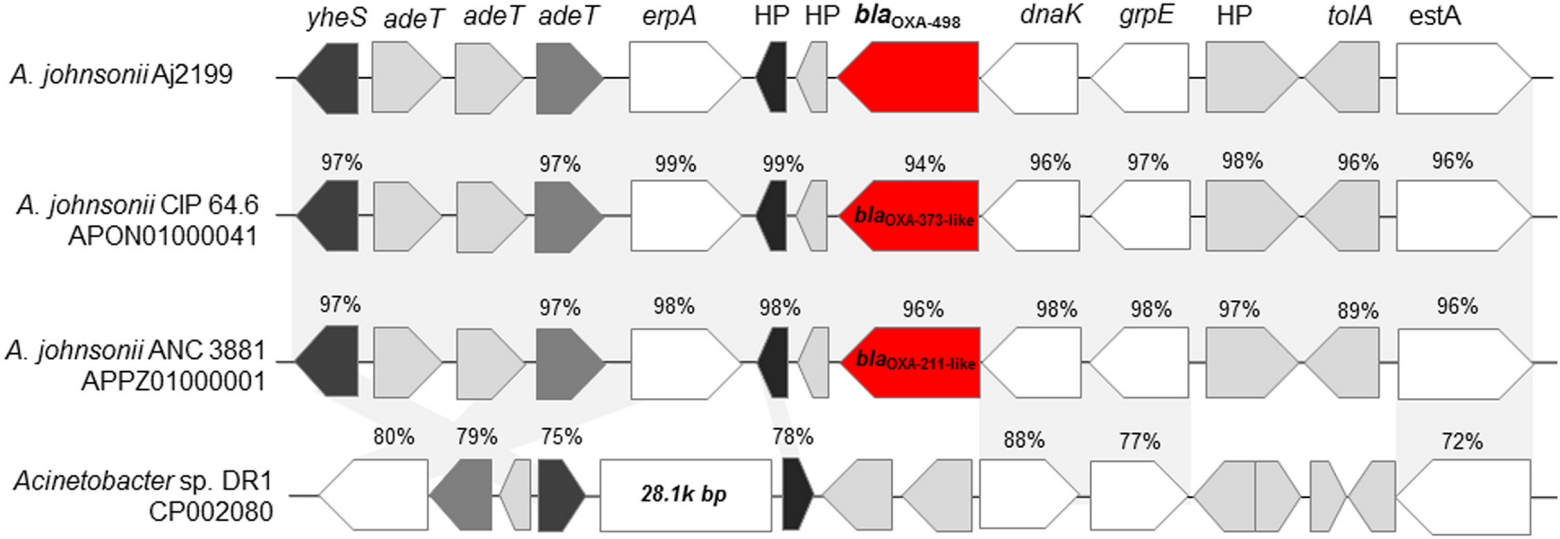
Genetic context of a new variant of *bla*_OXA-211_ in Aj2199. Analysis of the genetic context of *bla*_OXA-211_ and its variants.

The genetic context of OXA variants was conserved among all *A*. *johnsonii* strains (Fig 3). The region between *grpE* and *estA*, containing HP and *tolA*, is only conserved in *A*. *johnsonii*. Moreover, from our analysis, we observed *dnaK* with 75% identity in *Moraxella catarrhalis* BBH18 strain. However, *grpE* present in *M*. *catarrhalis* BBH18 did not show any significant similarities to those found in *A*. *johnsonii* strains.

A hypothetical protein (HP, shown in black in Fig 3), located downstream of *bla*_OXA-498_ is conserved in multiple *Acinetobacter* species. Farther downstream of *bla*_OXA-498,_ we found *erpA* and three different *adeT* genes, followed by *yheS*. The genetic context between *yheS* and the conserved HP is larger in other *Acinetobacter* sp. (ranging from 28.1 kbp to 29.7 kbp) compared to Aj2199 (3.9 kbp). The *yheS* gene is reversely oriented in *Acinetobacter* sp. DR1 compared to Aj2199 (Fig 3).

This analysis showed that the genes located upstream and downstream of *bla*_OXA-211_ and variants are conserved among *A*. *johnsonii* strains indicating the ubiquity of the gene.

### Resistance determinants present in Aj2199 and their genetic contexts supports the occurrence of HGT

To determine the repertoire of resistance genes present in Aj2199 genome we have used ARG-ANNOT database to predict them. A total of ten resistance gene sequences, excluding *bla*_OXA-498_, were identified. Among them we found genes encoding for β-lactamases (*bla*_PER-2,_ *bla*_OXA-58_ and *bla*_TEM -1_), aminoglycoside modification enzymes (*strA*, *strB* and *aacC2*), sulfonamide-resistant dihydropteroate synthase (*sul1*), erythromycin esterase (*ereA*) and macrolide efflux protein and phosphotranferase (*msrE* and *mphE*).

In order to analyze the organization and context of the β-lactamase genes, in-depth analysis of the genome sequences of Aj2199 was performed.

### Analysis of *bla*_OXA-58_ genetic context

**Fig 4:**
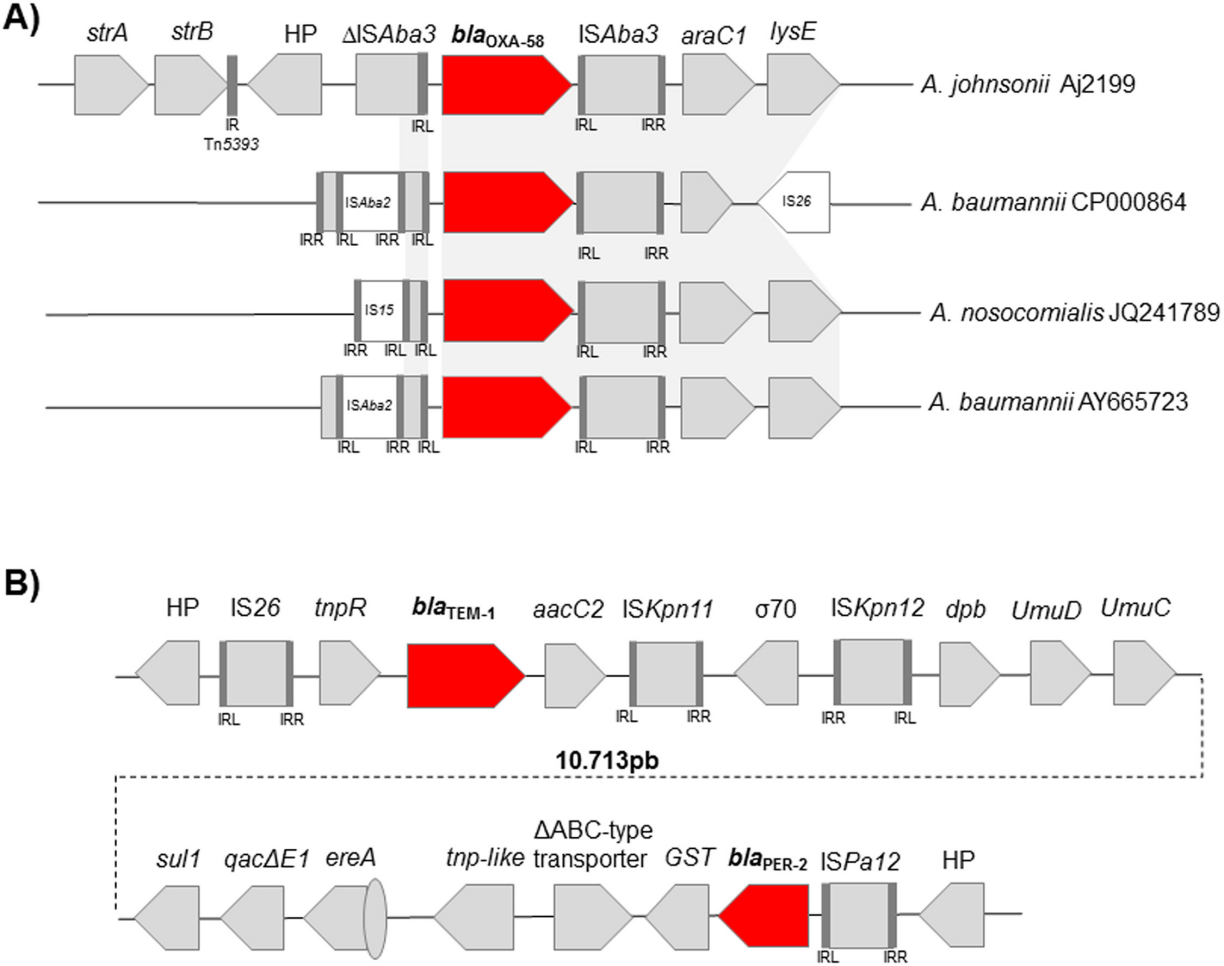
Resistance determinants present in Aj2199 and corresponding genetic contexts supports the occurrence of HGT. (A) Analysis of *bla*_OXA-58_ genetic context compared to other *bla*_OXA-58_ genetic contexts described. (B) Schematic representation of *bla*_PER-2_ *and bla*_TEM -1_ flanking regions.

The *bla*_OXA-58_ genomic context analysis showed the presence of a truncated IS*Aba3* upstream of the gene and a complete IS*Aba3* downstream of it (Fig 4A). The ΔIS*Aba3* located upstream (5’ end) has lost the right inverted repeat (IR-R). The presence of truncated versions of IS*Aba3* in the 5 ‘end of the *bla*_OXA-58_ gene were previously described by others showing that in some cases IS*Aba3* is interrupted by another IS, such as IS*Aba2* [38, 39] (Fig 4A). The genetic context downstream (3’ end) *bla*_OXA-58_ resembles the genetic structure previously described in *A*. *baumannii* strains and in the *A*. *nosocomialis* AG13TU119 strain [40] [39], where IS*Aba3* is followed by *araC1* and *lysE*. In contrast, the region flanking the 5’ end of the *bla*_OXA-58_ was not previously described. Directly upstream ΔIS*Aba3-bla*_OXA-58_ we found a hypothetical protein (HP) with 86% of identity to another HP described only in *Dendroctonus ponderosae* (GenBank accession number APGK01000113) and two streptomycin resistance determinants, *strB* and *strA*, are preceding it (Fig 4A). This region containing *strB* and *strA* is derived from Tn*5393* [41] and was previously described in some species such as *Erwinia amylovora*, *A*. *baumannii*, *Escherichia coli*, *Pseudomonas aeruginosa* (GenBank accession numbers M96392, CP010779, CP010373 and KP975076, respectively).

A blast analysis was performed and it showed that 62 *bla*_OXA-58_ sequences are available in the GenBank. However, the genetic context of *bla*_OXA-58_ was only described in 36 out of the 62 deposited sequences. Within the 36 descriptions, at least one copy of IS*Aba3* was associated to *bla*_OXA-58_ in the in the 5’ or 3’ end of the gene. The association of *bla*_OXA-58_ with IS*Aba3* seems to be a stable and successful platform for this carbapenemase.

Other genetic contexts, some of them described in plasmid, have been reported in the literature, e.g. the association of *bla*_OXA-58_ with a IS*Aba825*, IS*Aba2* and IS*18* upstream of *bla*_OXA-58_ [38, 39, 42–44].

A new genetic environment flanking the upstream (5’ end) region of the ΔIS*Aba3* was found in Aj2199 genome suggesting the acquisition of this gene from at least two different species.

### Genetic context analysis of *bla*_PER-2_ and *bla*_TEM-1_ exhibits the presence of foreign DNA acquisition

We analyzed the genetic context of *bla*TEM-1 in Aj2199 and found that *bla*TEM-1 is embedded in a truncated version of Tn*3*, in which the *tnpA* gene is absent and the *tnpR* gene is complete and shared 94%, 100% and 95% of identity to the previously *tnpR* described in the Tn*1*, Tn*2* and Tn*3* transposons, respectively [45]. Located downstream *bla*TEM-1 is *aacC2*, which has been shown to confer resistance to gentamicin (Fig 4B). The analysis of the contig (named contig_8) harboring *bla*TEM-1 demonstrated it was 24,769 bp away (from the 3’ end of the *bla*TEM-1 until the 3’ end of *bla*PER-2) from another important β-lactamases *bla*PER-2. Some of the genes located in the region between *bla*_TEM-1_ and *bla*_PER-2_ where previously described in the context of genomic islands (*umuC* and *umuD* genes) [46, 47], or in plasmids as the genes present in the Tn*21* mer operon [48].

Our analysis of the *bla*_TEM-1_ genetic context showed 99% identity to a previously described genetic context in *Klebsiella pneumoniae* (GenBank accession number LM994717). A complete IS*26* was located upstream of *tnpR* and preceded by HP that shared 97% identity with a HP described in an *A*. *pittii* (GenBank accession number WP_044100499) (Fig 4B).

Futhermore, the analysis of the genetic context of *bla*_PER-2_ showed that this gene was flanked by a complete IS*Pa12* upstream and a glutathione S-transferase downstream (Fig 4B). The association of this later gene with *bla*_PER-2_ was previously described in *Citrobacter freundii* (GenBank accession number AM409516) [49]. Preceding IS*Pa12* was a HP with 34% identity to a HP described in *Paraglaciecola polaris* (GenBank accession number WP_007103537). Moreover, a *top-*like gene with 97% identity to a transposes reported in *Methylophaga frappieri*–a bacteria typically isolated from marine environments or brackish waters–(GenBank accession number CP003380), was found 1,422 bp downstream of *bla*_PER-2._ This particular transposase was also identified in *Klebsiella oxytoca* (GenBank accession number CP003684) and *Vibrio salmonicida* (GenBank accession number AJ289135). Downstream this transposase in Aj2199 was a region containing a complete gene cassette (*ereA*), *qacEΔ1*, and *sul1*. The analysis of *bla*_TEM-1_ and *bla*_PER-2_ in Aj2199 indicates the occurrence of different recombination events. There was a great degree of mosaicism, suggesting an ability to acquire exogenous DNA from different species as well as exposing a high degree of genomic plasticity.

Since *bla*_TEM-1_ and *bla*_PER-2_ have been found to be located within plasmids or chromosomes [49–53], we performed various plasmid extraction techniques and used the products in transformation assays of competent *E*. *coli*, *A*. *baumannii* and *A*. *baylyi* cells. In addition, we carried out conjugation assays using different bacteria as host cells. Resistance could not be detected in any of the assays suggesting that *bla*_TEM-1_ and *bla*_PER-2_ are likely chromosomally located. Moreover, using a bioinformatic approach we searched for *Enterobacteriaceae* plasmid replicon sequences (n = 116) [30] and obtained negative results. In addition, as noted above, neither of these genes are included within the contigs predicted to be part of plasmid structures by the plasmidSpades software. Also, the *repAcil* PCR amplification gave negative results. In summary, although not definitive, the available evidence suggest that *bla*_TEM-1_ and *bla*_PER-2_ are not plasmid-mediated.

### Identification of potential prophages and phage sequences in Aj2199 genome

Using PHAST tool to predict phage sequences, we identified two intact prophages, a questionable prophage and an incomplete prophage in the Aj2199 genome.

One of the intact prophages contained 50,055 bp and a G+C content of 41.46% with 61 predicted open reading frames. This prophage has the corresponding *attL* (TATACAATGATTTTAG) and *attR* (TATACAATGATTTTAG) recognition sites. The phage integrase had a 99% identity with an integrase described in the genome of *A*. *johnsonii* (GenBank accession number WP_035326457) and an 83% identity with an integrase found in *A*. *bereziniae* genome (GenBank accession number WP_005029089). The genes required for the head, capsid, tail tape, tail fiber as well as the tail assembly protein encoding genes were present.

The second intact prophage contained 22,820 bp a G+C content of 43% with 29 predicted open reading frames. The genes encoding for putative head, capsid, tail tape and tail fiber were present. However, the *attL* and *attR* recognition sites as well as the integrase-encoding gene were not found.

Furthermore, a questionable prophage (24,313 bp), for which only phage proteins and transposases were recognized by PHAST, was identified.

The incomplete predicted prophage, composed of 21,414 bp, possessed 39 predicted open reading frames including the phage integrase, which had a 100% identity with an integrase in the *A*. *johnsonii* SH046 (GenBank accession number EEY97814). The *attL* (ATATCTAAGCAT) and *attR* (ATATCTAAGCAT) recognition sites were also present. The analyses of non-phage genes into the predicted prophage showed the presence of putative transposases and genes with unknown functions.

In agreement with previous published results, our findings revealed the presence of phage related sequences in the Aj2199 genome [54].

### Identification of insertion sequence elements in Aj2199 genome

The Aj2199 genome contains a large variety of insertion sequence (IS) elements, which reinforces the theory of extensive acquisition events of mobile genetic elements (MGEs) in this strain. A total of 19 complete ISs and 26 partial ISs were identified (Table 3). As was observed for other *Acinetobacter* sp., the most abundant IS elements within Aj2199 are IS*Aba*-like. Multiple copies of complete IS*Aba12* (n = 6), IS*Aba19* (n = 2), and IS*Aba8* (n = 2) were present in the genome. Furthermore, multiple copies of truncated ISs were found, including five copies of ΔIS*Aba19* and five copies of ΔIS*Aba11*. The IS*Aba12* and IS*Aba19*, in their complete and truncated versions, were the most abundant IS within Aj2199 genome. IS*Aba12* was found downstream or upstream of different genes in all instances. Genes found upstream of IS*Aba12* were involved in the synthesis of lipid A, which is known to be an activator of monocytes triggering a proinflammatory response and is recognized as a Toll-like receptor 4, TLR4 [55, 56]. A copy of IS*Aba12* was found downstream of Ton-B dependent receptor gene, which codes for bacterial outer membrane proteins that transports siderophores, vitamin B12, nickel complexes, and carbohydrates [57]. We also observed other copies of IS*Aba12* in different genetic context, e.g. near to hypothetical proteins or putative genes related to zinc metabolism or UV radiation resistance.

**Table 3:** Insertion sequences identified in *A*. *johnsonii* Aj2199 genome.

Insertion sequences	Origin	Complete/partial sequences	Amount
IS*26*	*Proteus vulgaris*	1/0	1
IS*Aba2*	*A*. *baumannii*	0/1	1
IS*Aba3*	*A*. *baumannii*	1/1	2
IS*Aba5*	*A*. *baumannii*	0/1	1
IS*Aba7*	*A*. *baumannii*	0/1	1
IS*Aba8*	*A*. *baumannii*	2/0	2
IS*Aba11*	*A*. *baumannii*	0/5	5
IS*Aba12*	*A*. *baumannii*	6/4	10
IS*Aba14*	*A*. *baumannii*	1/0	1
IS*Aba19*	*A*. *baumannii*	2/5	7
IS*Aba21*	*A*. *baumannii*	0/1	1
IS*Aba25*	*A*. *baumannii*	0/1	1
IS*Aba31*	*A*. *baumannii*	0/1	1
IS*Aha2*	*A*. *haemolyticus*	1/0	1
IS*Ajo1*	*A*. *johnsonnii*	1/0	1
IS*Ajo2*	*A*. *johnsonnii*	0/1	1
IS*Kpn11*	*Klebsiella pneumoniae*	1/0	1
IS*Kpn12*	*Klebsiella pneumoniae*	1/0	1
IS*Kpn18*	*Klebsiella pneumoniae*	0/1	1
IS*Our1*	*Oligella urethralis*	1/0	1
IS*Pa12*	*Pseudomonas aeruginosa*	1/0	1
IS*Pa14*	*Pseudomonas aeruginosa*	0/1	1
IS*Pst3*	*Pseudomonas stutzeri*	0/1	1
IS*Swi1*	*Salmonella enterica*	0/1	1

Additionally, IS elements previously described in *K*. *pneumoniae* such as IS*Kpn11*, IS*Kpn12* and IS*Kpn18* were identified. IS*Kpn11* and IS*Kpn12* were found flanking the RNA polymerase σ^70^ factor (*rpoD*), which may allow for the movement of *rpoD* within the genome as well to be transferred to another bacterial cell. This σ^70^ factor was also described in plasmids and in the chromosome of many other species, such as *K*. *pneumoniae*, *A*. *baumannii*, *E*. *coli*, *Serratia marcescens* and *Shigella sonnei* [58].

IS*Our1* was also found in Aj2199 genome. This IS, which has been previously reported as a strong promoter when localized upstream of *ampC* in *Oligella urethralis* [59], is found upstream of a putative gene belonging to an UvrABC system.

The present data exposed the importance of ISs in modeling Aj2199 genome and its ability to acquire and later recombine exogenous DNA within its genome.

### Presence of putative RNA regulators in Aj2199 genome

**Fig 5:**
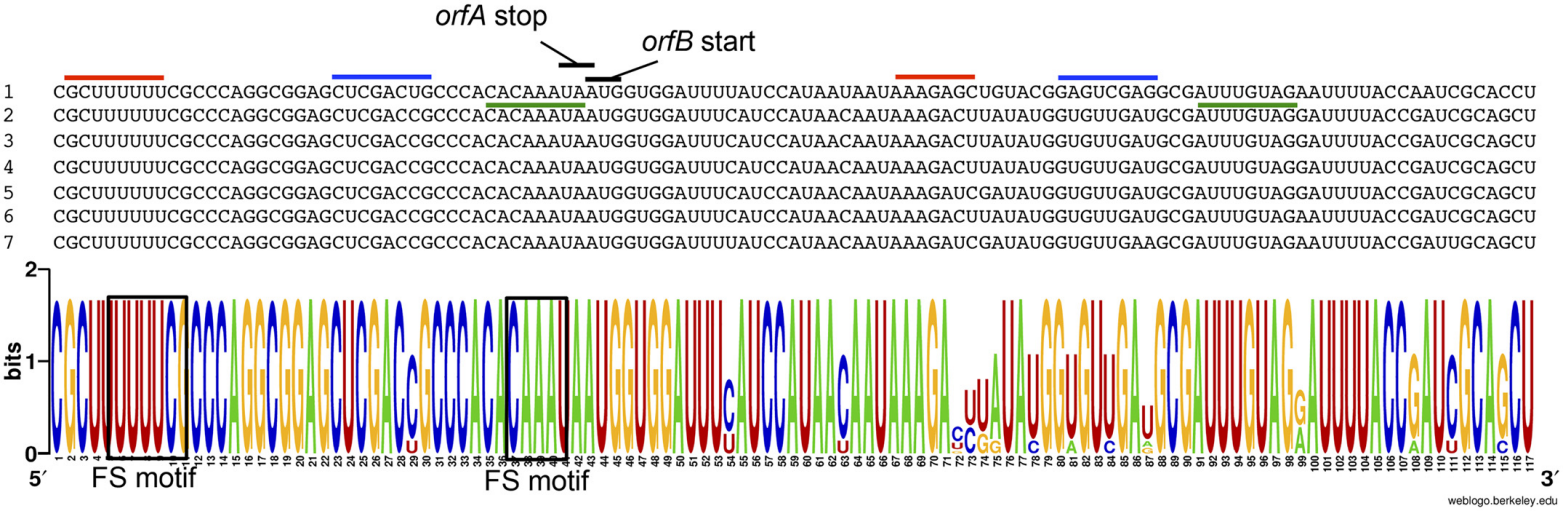
Conserved sequence of ALIL-pseudoknot associated to IS*Aba* insertion sequences. Sequences involved in the predicted RNA secondary structure are indicated with red and blue lines. Putative pseudoknot pairing is indicted with green lines. Frame-shift motives are marked with black squares.

*In-silico* prediction of putative regulatory RNAs, including small RNA, antisense RNA, thermoregulators, riboswitches and cis-regulators, as well as other small non-coding RNA involved in cellular processes, such as CRISPRs and antitoxins was assessed in the Aj2199 genome. To perform the search we used 720 covariance models resulting in 66 potential candidates. Among them, twenty-eight showed significant e-values corresponding to four riboswitches (families FMN, TPP, cobalamin, glycine), one thermoregulator (CspA), 13 sRNA (CsrC, MicC, t44, RsmZ, RsmY, IsrG, IsrK, SraL, rli40, STnc40, STnc370, Aar, sau-50, Atu_C9), one asARNA (C4) and four cisRNA (yybP-ykoY, Alpha_RBS, ykkC-yxkD, ALIL pseudoknot). While most of these candidates have previously been studied [60, 61], the role of many such as the apical loop-internal loop RNA (ALIL) pseudoknot is still unclear and poorly understood, and thus was further analyzed in this study. In particular, the apical loop-internal loop RNA (ALIL) pseudoknot, was further analyzed. This RNA is directly involved in the stimulation of transposition in IS*3*-like elements [62]. RNA prediction of ALIL pseudoknot retrieved seven positive sequences that were located within an IS (Fig 5). Five of them were part of an IS*Aba19* and the remaining pseudoknots were found in IS*Aba2*. Sequence analysis of the pseudoknot sequences showed a highly conserved sequence with two main palindromic regions that are marked with red and blue lines in Fig 5. In Fig 5 the base pairing interaction involved in the pseudoknot was marked with green lines. Noteworthy, IS*Aba19* was the second most predominant after IS*Aba12* within Aj2199 genome. The role of the ALIL regulatory RNA in *A*. *johnsonii* in the activity of IS*Aba19* will be studied in future studies.

Our analysis exposed a wide variety of regulatory elements in Aj2199 genome. The presence of several MGEs and RNA regulators within this genome suggests a potential contribution of MGEs in the mobilization of RNA elements that have an impact on genome evolution and bacteria adaptation.

### Dispersion of resistance determinants in other *A*. *johnsonii* clinical strains

In order to determine if other *A*. *johnsonii* isolates recovered from the same hospital harbored some of the resistance determinants present in Aj2199, we tested four *A*. *johnsonii* strains (Aj205, Aj286, Aj289, Aj306) (Table 1). Among the tested isolates Aj286 and Aj289 were resistant to FOX. Moreover, Aj286 was resistant to AMP and SAM. The other two isolates, Aj205 and Aj306, were susceptible to all antibiotics tested (Table 1). PCR amplification for *bla*_PER-2_, *bla*_OXA-58_, *bla*_TEM-1_, *strA*, *strB*, *ereA*, *sul1*, and *aacC2* were performed. Positive amplification results were only obtained for *strA* and *strB* in Aj289, Aj286, and Aj306.

## Discussion

In the last few years, the increase of antibiotic resistant bacteria has posed a serious threat to human health. The recoveries of species not previously recognized as a menace in the hospital environment have been widely reported [63–65].

In the present study we have delved into the genome of a multidrug-resistant *A*. *johnsonii* strain (Aj2199) that was recovered from a hospital in Buenos Aires and it exhibited the occurrence of resistance genes previously reported in other species. By the implementation of different bioinformatics tools, we analyzed in depth its genome to identify particular features and the presence of HGT events within it.

A wide variety of MGEs and traces of HGT were identified in Aj2199. These results are in accordance with previous studies that reported great number of MGEs in *Acinetobacter* genomes [42, 54, 66–69]. Ten resistance genes (*bla*_PER-2_, *bla*_OXA-58_, *bla*_TEM-1_, *strA*, *strB*, *ereA*, *sul1*, *aacC2*, *msrE* and *mphE*) were identified demonstrating the potential of other non-*baumannii Acinetobacter* species to evolve towards the development of high levels of antibiotic resistance. Moreover, a new variant of *bla*_OXA-211,_ called *bla*_OXA-498_, was also identified reinforcing the idea that some *Acinetobacter* sp. harbor a chromosomally encoded oxacillinase. *bla*_OXA-498_ has up to 72% amino acid identity with other oxacillinases described in *Acinetobacter* species. This supports the uniqueness of the OXA variant to *A*. *johnsonii*.

Regarding other relevant β-lactamases present in Aj2199 genome, analysis of *bla*_PER-2_ and *bla*_TEM-1_ genetic contexts revealed a great degree of mosaicism evidencing its ability to acquire exogenous DNA from different species as well as exposing a high degree of genomic plasticity. The presence of genetic contexts previously described in the *Enterobacteriaceae* family supported the occurrence of DNA exchange within them. The analysis of *bla*_OXA-58_ genetic environment exposed that the flanking region upstream (5’ end) the ΔIS*Aba3* was novel. The 3’ end resembled the genetic environment previously described in other *Acinetobacter* strains [40] [39].

The results obtained here showed that *bla*_OXA-58_, *bla*_PER-2_, and *bla*_TEM-1_ are likely chromosomally located. This differs from a recent study where the presence of different β-lactamase genes, such as *bla*_NDM-1_, *bla*_OXA-58_ and *bla*_PER-1,_ were plasmid located [50].

The role of IS in the development of antibiotic resistance and genome plasticity in *A*. *baumannii* strains has been extensively mentioned in the literature [69, 70]. However, the role, quantity, and identity of ISs in other species have been poorly studied. In the Aj2199 genome a large number of complete or partial IS were found. A total of 45 IS-related sequences belonging to different families were identified.

IS elements previously described in *K*. *pneumoniae* such as IS*Kpn11*, IS*Kpn12* and IS*Kpn18* were identified, which reinforces the idea of extensive acquisition of mobile genetic elements and the importance of ISs in modeling Aj2199 genome and its ability to acquire and later recombine exogenous DNA within its genome.

Different phages, prophages and phage related proteins have been found in *A*. *baumannii*, as well as in other species of this genus [54, 71, 72]. Touchon *et al*. found that temperate phages account for a great proportion of *Acinetobacter’s* genome identifying over 260 prophages among the genomes in their study [54]. It is well known that phages play a crucial role in HGT as well as in the evolution and dynamics of bacterial genomes [54, 73]. There are multiple descriptions of phage related genes, cryptic prophages and prophage structures in *Acinetobacter* sp. [54, 71, 72, 74]. However, the role of phages in *Acinetobacter* is poorly understood. We have identified two intact phages and other phages related sequences within Aj2199 genome, supporting the general idea that phages impact in the evolution of bacterial genomes.

To the best of our knowledge, RNA regulatory sequences have not yet been identified in *A*. *johnsonii*. Only one article has addressed the role of regulatory RNAs in *Acinetobacter* specifically *A*. *baylyi* [75]. In that manuscript the Aar RNA regulator was described, which is an RNA element involved in amino acid metabolism [75]. Noteworthy, *A*. *johnsonii* Aj2199 also has a putative Aar regulator along with several other regulatory elements. In addition, it encodes the ALIL regulatory RNA, which we found within the second most abundant IS distributed in *A*. *johnsonii* Aj2199 genome. Future studies to address its function(s) will be conducted.

The great number of MGEs in the Aj2199 genome suggests a direct impact in the evolution and adaptability of this strain to extreme environment due to the fact that it showed an increasing number of antibiotic resistance determinants within it genome.

The data presented here strongly suggested that *A*. *johnsonii* actively acquires exogenous DNA from other bacterial species and concomitantly becomes a reservoir of resistance genes.

## Supporting Information

S1 Table Strains sequences analyzed in this study. (XLSX)

S2 Table List of homologous genes exclusively found in *A*. *johnsonii* genomes when comparing with the other *Acinetobacter* genomes (see S1 Table for a complete list of genomes used). GO term is added next to each gene family as functional annotation. Contig and position of each Aj2199 specific gene is also indicated.
(XLSX)

S3 Table List of gene potentially found in *A*. *johnsonii* AJ2199 plasmids according to plasmidSPAdes software prediction. (XLSX)

S4 Table Two-way average nucleotide identity (Two-way ANI) estimated among closely related strains of the *Acinetobacter* sp. AJ2199. (XLSX)
